# Osthole attenuated cytotoxicity induced by 6-OHDA in SH-SY5Y cells through inhibition of JAK/STAT and MAPK pathways

**DOI:** 10.22038/IJBMS.2023.68292.14905

**Published:** 2023

**Authors:** Samira Barangi, Parisa Hosseinzadeh, Gholamreza Karimi, Zahra Tayarani Najaran, Soghra Mehri

**Affiliations:** 1 Pharmaceutical Research Center, Pharmaceutical Technology Institute, Mashhad University of Medical Sciences, Mashhad, Iran; 2 Department of Pharmacodynamics and Toxicology, School of Pharmacy, Mashhad University of Medical Sciences, Mashhad, Iran; 3 Medical Toxicology Research Center, Mashhad University of Medical Sciences, Mashhad, Iran

**Keywords:** 6-OHDA, Apoptosis, JAK Kinases, Mitogen-activated protein-kinase, Osthole, STAT3

## Abstract

**Objective(s):**

Natural coumarin called osthole is regarded as a medicinal herb with widespread applications in Traditional Chinese Medicine. It has various pharmacological properties, including antioxidant, anti-inflammatory, and anti-apoptotic effects. In some neurodegenerative diseases, osthole also shows neuroprotective properties. In this study, we explored how osthole protects human neuroblastoma SH-SY5Y cells from the cytotoxicity of 6-hydroxydopamine (6-OHDA).

**Materials and Methods:**

Using the MTT assay and DCFH-DA methods, respectively, the viability of the cells and the quantity of intracellular reactive oxygen species (ROS) were evaluated. Signal Transducers and Activators of Transcription (STAT), Janus Kinase (JAK), extracellular signal-regulated kinase 1/2 (ERK1/2), c-Jun N-terminal kinase (JNK), and caspase-3 activation levels were examined using western blotting.

**Results:**

In SH-SY5Y cells, the results showed that a 24-hour exposure to 6-OHDA (200 µM) lowered cell viability but markedly elevated ROS, p-JAK/JAK, p-STAT/STAT, p-ERK/ERK, p-JNK/JNK ratio, and caspase-3 levels. Interestingly, osthole (100 µM) pretreatment of cells for 24 hr prevented 6-OHDA-induced cytotoxicity by undoing all effects of 6-OHDA.

**Conclusion:**

In summary, our data showed that osthole protects SH-SY5Y cells against 6-OHDA-induced cytotoxicity by inhibiting ROS generation and reducing the activity of the JAK/STAT, MAPK, and apoptotic pathways.

## Introduction

Currently, Parkinson’s disease (PD) is considered the most common and chronic age-related neurodegenerative disorder with an effect on almost 2% of people worldwide and a prevalence of 5% over the age of 85 (1). In PD, the progressive neurological condition occurs because dopaminergic neurons selectively degenerate in the dorsal part of the substantia nigra and lead to abnormal motor control due to decreased levels of dopamine in the striatum (2). Different mechanisms, including oxidative stress, mitochondrial dysfunction, neuroinflammation, and apoptosis of dopaminergic neurons, are involved in the pathogenesis of PD (3), although the principle mechanism of initiation of PD has stayed unclear.

Several models have been used to create a pathophysiological process very similar to PD. Among the different neurotoxic models of PD, the 6-hydroxydopamine (6-OHDA) is widely applied for different models of PD research (4). 6-OHDA is a crucial exogenous neurotoxin formed by dopamine oxidation and it is beneficial for the neurodegeneration studies like PD because of the induction of dopaminergic neuronal degradation (5). 6-OHDA causes dopaminergic neurotoxicity by accumulation in nigral neurons (3). One of the main mechanisms of 6-OHDA toxicity can involve the reactive oxygen species generation (ROS) during its autoxidation process. Once inside the cell, it rapidly oxidizes and generates ROS, which impairs mitochondrial function and ultimately causes neuroinflammation and damage to dopaminergic neurons (6, 7).

Oxidative stress is an important reason for cellular toxicity in the central nervous system and promotes inflammation and apoptosis in neurodegenerative disorders like PD (8). Hence, understanding the cellular mechanisms that increase neuronal resistance to oxidative stress may prepare new avenues for PD treatment. Different molecular signaling such as JAK2/STAT3 and MAPK pathways are involved in the inflammation and apoptosis pathway regulation in PD (9, 10).

The Janus Kinase/Signal Transducers and Activators of Transcription (JAK/STAT) is a regulatory pathway, which activated by numerous cytokines, interferons, and growth factors (11), is involved in cell survival, proliferation, angiogenesis, inflammation, and apoptosis (12, 13). Aberrant activation of JAK/STAT is apparent in neuroinflammation and neurodegenerative diseases like Multiple Sclerosis, Alzheimer’s, and PD (10). 

The mitogen-activated protein kinase (MAPK), a serine/threonine protein kinases superfamily, is responsible to regulate various intracellular signaling in Eukaryotes including cell differentiation and proliferation, apoptosis or survival, inflammation, and innate immunity (14). C-Jun N-terminal kinase (JNK), extracellular signal-regulated kinases (ERK), and p38 kinase, as the main groups of MAPK, are particularly important in PD (9) and considered apoptosis factors which activate through oxidative stress (15). JNK and p38 have a substantial task in neuronal damage, and ERK over-activation is known to contribute to dyskinesia in PD striatum (16, 17). The oxidative stress in PC12 cells created by 6-OHDA leads to enhanced JNK phosphorylation and increases cell apoptosis (18, 19).

Osthole (7-Methoxy-8-(3-methylbut-2-enyl)-2-chromenone)

is a coumarin derivative of a natural plant, firstly obtained from *Cnidium* *Monnieri* (20). It is found in various medicinal plants like the mature fruit of *C. monnieri* with an osthole high content, which is used in Traditional Chinese Medicine (21). In several experimental studies, the pharmacological effects of osthole including anticancer (22), anticonvulsant (23), hepatoprotective (24), cardiovascular protective (25), and neuroprotective (26) have been reported. Moreover, osthole has a potential anti-oxidant effect as well (27, 28). Osthole exerts physiological effects through regulation of various signaling pathways including JAK/STAT and MAP kinase which in turn modulate cell cycle regulators, transcriptional factors, and proliferation (28, 29).

Osthole indicated neuroprotection opposite to many neurodegeneration experimental models. For instance, osthole relieved the symptoms of Alzheimer’s disease by attenuating inflammation and oxidative stress (30, 31). Moreover, osthole reduced oxidative stress and suppressed inflammation and apoptosis in various models of PD (26, 32). Besides, previous studies have found that osthole has a protective effect on different tissues through the regulation of MAPK and JAK/STAT3 signaling pathways (27, 33).

According to previous studies, oxidative stress, as well as apoptosis, are two main mechanisms of neurotoxicity created by 6-OHDA. Therefore, this research aimed to investigate the effect of osthole on neurotoxicity by 6-OHDA in the SH-SY5Y cell line by focusing on the activity of apoptosis, and JAK/STAT and MAPK pathways.

## Materials and Methods

***Materials***

Osthole was obtained from Golexir Pars (Iran). 6-OHDA, MTT reagent, the fluorescent probe DCFH-DA, and penicillin-streptomycin (PS) were bought from Sigma (Germany). DMEM/F12 medium was purchased from Bio-Idea (Iran) and FBS from Gibco (USA). In this study, all used antibodies were obtained from Cell Signaling (USA).

***Cell culture and treatment***

The human neuroblastoma SH-SY5Y cell line was procured from the Pasteur Institute (Iran). The culture medium DMEM-F12 with 10% FBS along with penicillin and streptomycin were applied to culture cells and maintained in a 37 °C incubator with 95% humidity containing 5% CO_2_ concentration and passaged at 80% confluence.

***Cell viability assay***

SH-SY5Y cells were seeded in a microplate with 96 wells (10^4^ cells/well). Cell viability was evaluated by 6-OHDA exposure (24 hr), using an MTT assay to determine the IC_50_ value. Moreover, the effect of osthole in different concentrations on cell viability was determined after 48 hr exposure. For this purpose, cells were treated with 6-OHDA in different concentrations (50–200 μM) and different concentrations (25–500 μM) of osthole for 24 hr and 48 hr, respectively. Afterward, the efficacy of osthole on cytotoxicity by 6-OHDA in SH-SY5Y cells was tested. Briefly, cells (10^4^ cells/well) were exposed to a different concentration of osthole (25–500 μM) for 24 hr and then incubated with 6-OHDA (200 μM; IC_50_). After 24 hr, the MTT solution at the final concentration of 0.5 mg/ml was poured on each well of the 96-well culture plate, and then the plate was put into a 37 °C incubator for 3 hr. After the removal of the upper culture medium, the insoluble purple formazan crystal was dissolved in 150 μl of dimethylsulfoxide (DMSO) which created a color solution, with maximum absorbance at 545 nm measurable by a microplate reader (Start Fax-2100, UK).

***Measurement of intracellular ROS generation***

To determine the amount of ROS in SH-SY5Y cells, we used the DCFH-DA (2′,7′-dichlorofluorescein diacetate) method based on the Li *et al*. study with some modification (34). DCFH-DA reagent is a fluorimetric probe, which can enter the cell and deacetylate to DCFH with intracellular esterases. In the vicinity of ROS, DCFH can convert to DCF with high fluorescence. For this test, 10^4^ SH-SY5Y cells/well were seeded in 96-well microplates and incubated with osthole (25–500 μM) for 24 hr before treatment by 6-OHDA (200 μM). Twenty four hours later, the upper mediate was removed and 10 μM DCFH-DA was poured into the cultured cells for 30 min in a 37 °C incubator. After cells were washed with PBS, ROS generation was evaluated by the fluorescence intensity of DCF (excitation and emission wavelengths at 485 and 538 nm, respectively).

***Western blot analysis***

To investigate whether osthole had a protective effect against 6-OHDA, the expressions of proteins including caspase-3, JAK2, p-JAK2, STAT3, p-STAT3, ERK, p-ERK, JNK, and p-JNK were identified by western blot. For this purpose, About 10^6^ SH-SY5Y cells were exposed to 6-OHDA (200 μM) 24 hr after osthole (100 μM). The cells were collected, and after washing with cold PBS, cells were lysed in a lysis buffer according to the instructions previously published (35). Protein concentration was ascertained by applying the Bradford assay. Then western blot test was performed regarding the protocol of our previous study (36). In a nutshell, 10 µl of samples were loaded and electrophoresed on SDS polyacrylamide gel. Proteins were transferred to PVDF membranes, which were blocked by 5% skimmed milk or BSA (Bovine Serum Albumin) for non-phosphorylated or phosphorylated proteins, respectively, at room temperature (2 hr). In the next step, the membranes were exposed to rabbit monoclonal antibodies against caspase-3 (#9665), JAK2 (#3230), p-JAK2 (#3776), STAT3 (#12640), p-STAT3 (#9145), ERK (#4695), p-ERK (#9106), JNK (#9252), p-JNK (#9255), and mouse monoclonal antibody against β-actin (#3700), as primary antibodies, and IgG labeled with horseradish peroxidase (anti-rabbit or anti-mouse), as secondary antibodies. To visualize protein bands, enhanced chemiluminescence was used and the bands’ optical densities were measured by Gel doc (Alliance 4.7, UK). The densitometric analysis of bands was calculated by UVtec software (UK) and normalized to the related β-actin.

***Statistics analysis***

Prism 7.0 software (GraphPad Software, La Jolla, CA, United States) was used to analyze all data. The results were displayed as means ± SD. Statistical comparisons were evaluated by one-way analysis of variance test (ANOVA) followed by the Tukey–Kramer *post hoc* test. All experiments were repeated in triplicate, and *P*-value <0.05 was considered the significant level. 

## Results

***Effect of 6-OHDA on cell viability in SH-SY5Y cells***

**Figure 1:**
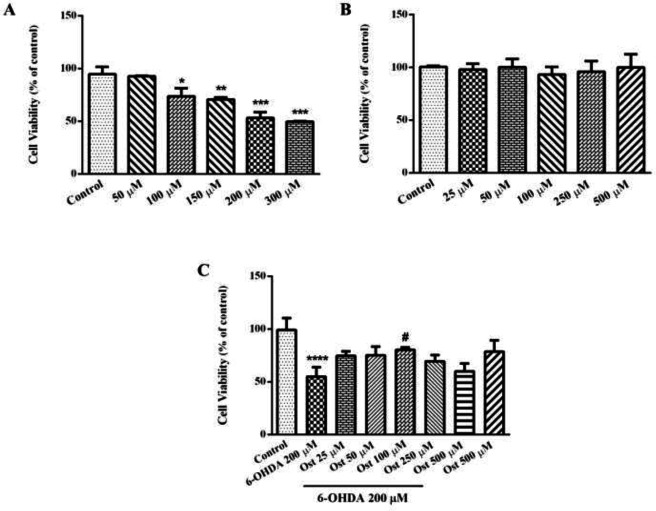
Effect of 6-OHDA (50–300 μM for 24 hr A), osthole (25–500 μM for 48 hr; B), and the effect of osthole (Ost) on 6-OHDA-induced cytotoxicity for 24 hr pretreatment (C) on SH-SY5Y cell viability by the MTT assay

The cell cytotoxicity of the 6-OHDA was measured by MTT assay to determine the IC_50 _of 6-OHDA. As shown in Figure 1A, 6-OHDA in 24 hr treatment decreased the viability of cells in comparison to the control group. IC_50_ of 6-OHDA after 24 hr exposure was 200 ± 17.5 μM.

***Effect of osthole on cell viability in SH-SY5Y cells***

The SH-SY5Y cells were exposed to osthole at different concentrations (25–500 μM) for 48 hr. Osthole in none of the concentrations had any significant effect on cell viability compared with the untreated group (Figure 1B).

***Effect of osthole on 6-OHDA-induced cytotoxicity in SH-SY5Y cells***

The results in Figure 1C indicated the decline in cell viability due to 6-OHDA (200 μM) exposure for 24 hr as compared to untreated cells (*P*<0.0001), while the pretreatment with 100 μM osthole for 24 hr markedly enhanced cell viability (*P*<0.05). In addition, no significant effect on cell viability was observed in pretreatment by other concentrations of osthole (Figure 1C).

***Effect of osthole on ROS production by 6-OHDA in SH-SY5Y cells***

**Figure 2:**
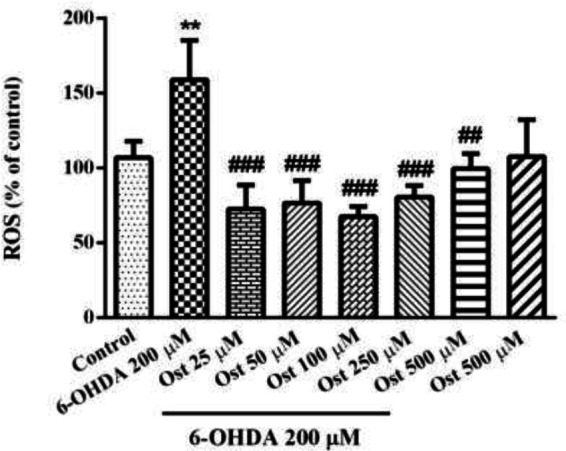
Effect of osthole (Ost) for 24 hr pretreatment on 6-OHDA-induced ROS production in SH-SY5Y cells by using the DCFH-DA reagent

6-OHDA treatment significantly enhanced the intracellular ROS in SH-SY5Y cells (*P*<0.01). Interestingly, the 24 hr exposure of cells with osthole (25–500 μM) before exposure to 6-OHDA markedly inhibited ROS production. These results showed a protective effect of osthole against cytotoxicity induced by 6-OHDA (Figure 2).

***Effect of osthole and 6-OHDA on JAK2/STAT3 proteins level in SH-SY5Y cells***

**Figure 3:**
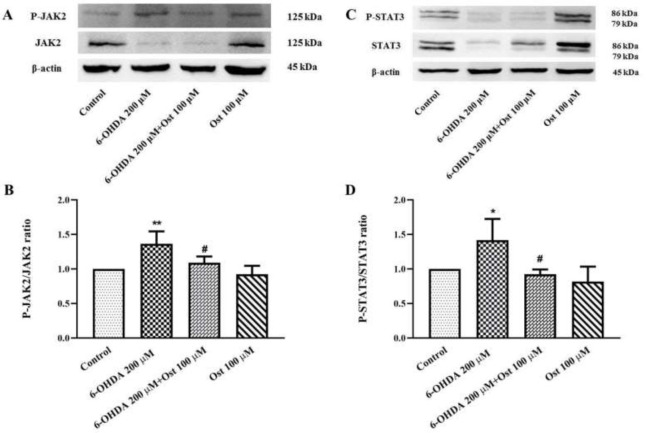
Effect of osthole (Ost) for 24 hr pretreatment and 6-OHDA for 24 hr on protein level of JAK2, phospho(P)-JAK2, STAT3, and phospho(P)-STAT3 in SH-SY5Y cells

The results showed that 6-OHDA exposure for 24 hr significantly increased the p-JAK2/JAK2 ratio in SH-SY5Y cells (*P*<0.01). In return, the pretreatment of the cells by osthole for 24 hr reduced the p-JAK2/JAK2 ratio in SH-SY5Y cells compared to the 6-OHDA group (*P*<0.05). Although the protein levels of p-STAT3 and STAT3 were separately reduced after 24 hr treatment with 6-OHDA in SH-SY5Y cells compared with control, the p-STAT3/STAT3 ratio enhanced in comparison to the untreated group (*P*<0.05) (Figure 3C and D). Moreover, the results demonstrated that 24 hr pretreatment of cells by osthole could decrease the p-STAT3/STAT3 ratio against the 6-OHDA group (*P*<0.05) (Figure 3C and D).

***Effect of osthole and 6-OHDA on the MAP kinase pathway proteins activity (JNK and ERK) in SH-SY5Y cells***

**Figure 4:**
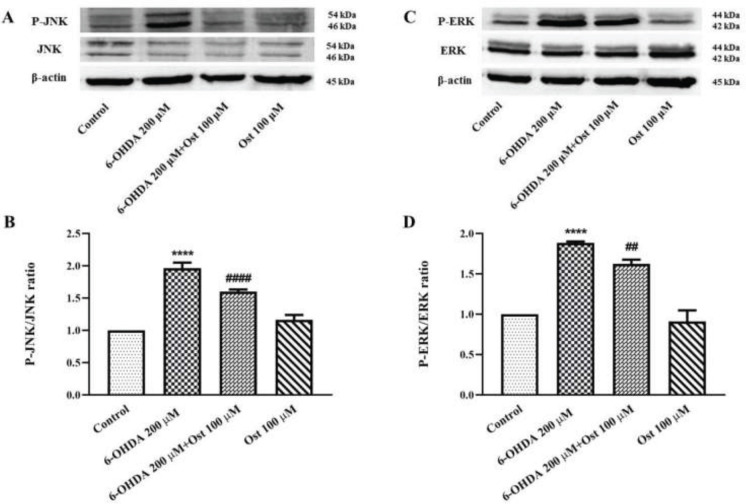
Effect of osthole (Ost) for 24 hr pretreatment and 6-OHDA for 24 hr on the protein level of JNK, phospho(P)-JNK, ERK, and phospho(P)-ERK in SH-SY5Y cells

The results demonstrated that after cell treatment with 6-OHDA, the p-JNK/JNK ratio elevated (*P*<0.0001), whereas 24 hr pretreatment with osthole attenuated the p-JNK/JNK ratio (*P*<0.0001). Furthermore, 24 hr exposure of 6-OHDA at a concentration of 200 μM strikingly induced the p-ERK/ERK ratio in SH-SY5Y cells in comparison to control cells (*P*<0.0001), while pretreatment of cells by osthole (100 μM) reduced the p-ERK/ERK ratio compared to the 6-OHDA group (*P*<0.01) (Figure 4C and D).

***Effect of osthole and 6-OHDA on the expression of caspase-3 in SH-SY5Y cells***

**Figure 5:**
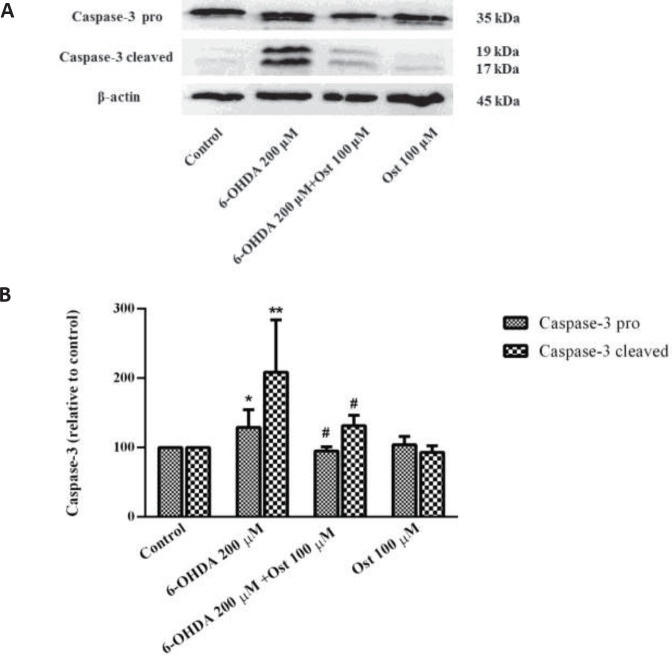
Effect of osthole (Ost) for 24 hr pretreatment and 6-OHDA for 24 hr on the protein level of caspase-3 pro and cleaved in SH-SY5Y cells

As indicated in Figure 5, the caspase-3 level (pro and cleaved) enhanced in SH-SY5Y cells when exposed to 6-OHDA compared to the untreated group (*P*<0.05 and *P*<0.01, respectively), while pretreatment of osthole at a 100 μM concentration reduced the pro and cleaved caspase-3 compared to the 6-OHDA treated cells (*P*<0.05).

Data are expressed as means ± SD. Data were analyzed by one-way ANOVA following the Tukey-Kramer post-test for multiple comparisons. *P<0.05, **P<0.01, ***P<0.001, and ****P<0.0001 vs control group, #*P*<0.05 vs 6-OHDA treated group

Ost: Osthole, 6OHDA: 6-ydroxydopamine

Data are expressed as means ± SD. Data were analyzed by one-way ANOVA following the Tukey-Kramer post-test for multiple comparisons. **P<0.01 vs control group, ##*P*<0.01 and ####*P*<0.0001 vs 6-OHDA group

Ost: Osthole, 6OHDA: 6-ydroxydopamine

The blots (A and C) and the bars (B and D) exhibit the densitometry analysis of western blots for p-JAK2/JAK2 and p-STAT3/STAT3 ratio. Equal loading of proteins is illustrated by β-actin bands. Data are expressed as means ± SD. Data were analyzed by one-way ANOVA following the Tukey-Kramer post-test for multiple comparisons. *P<0.05 and **P<0.01 vs control group, #*P*<0.05 vs 6-OHDA group

Ost: Osthole, 6OHDA: 6-ydroxydopamine

The blots (A and C) and the bars (B and D) exhibit the densitometry analysis of western blots for p-JNK/JNK and p-ERK/ERK ratio. Equal loading of proteins is illustrated by β-actin bands. Data are expressed as means ± SD. Data were analyzed by one-way ANOVA following the Tukey-Kramer post-test for multiple comparisons. ****P<0.0001 vs control group, ##*P*<0.01 and ####*P*<0.0001 vs 6-OHDA group

Ost: Osthole, 6OHDA: 6-ydroxydopamine

The blots (A) and the bars (B) exhibit the densitometry analysis of western blots for the level of caspase-3 pro and cleaved. Equal loading of proteins is illustrated by β-actin bands. Data are expressed as means ± SD. Data were analyzed by one-way ANOVA following the Tukey-Kramer post-test for multiple comparisons. *P<0.05 and **P<0.01 vs control group, #*P*<0.05 vs 6-OHDA group

Ost: Osthole, 6OHDA: 6-ydroxydopamine

**Figure 6:**
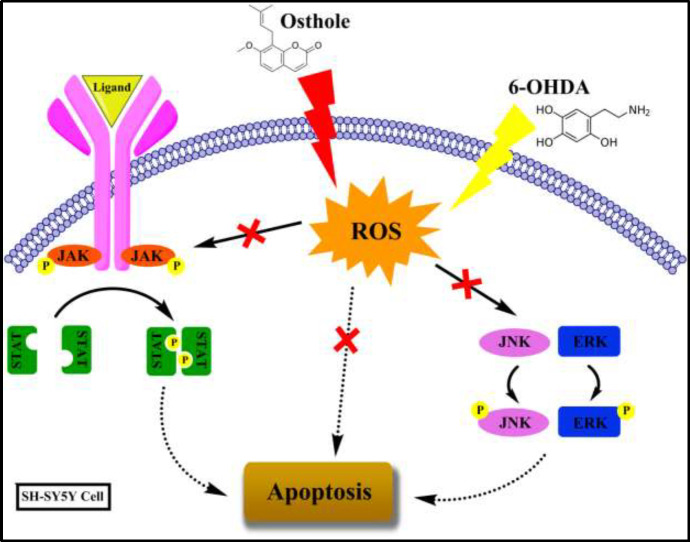
Mechanism of 6-OHDA in inducing apoptosis through JAK/STAT and MAPK pathways in SH-SY5Y cell and the role of osthole in preventing neuronal apoptosis

## Discussion

This research investigated the osthole protective effects on cytotoxicity in SH-SY5Y cells treated by 6-OHDA through JAK/STAT, MAPK, and apoptosis signaling pathway. Our results exhibited that 6-OHDA exposure induced oxidative stress and apoptosis through the increased intracellular ROS and caspase-3 levels and the phosphorylated form of JAK2, STAT3, JNK, and ERK proteins in SH-SY5Y cells. In contrast, the pretreatment of the cells with osthole reversed all changes caused by 6-OHDA in SH-SY5Y cells. It seems that osthole protects SH-SY5Y cells against 6-OHDA cytotoxicity.

Oxidative stress, apoptosis as well as inflammation lead to the loss of dopaminergic neurons which are important pathologies for PD (32). Numerous studies demonstrated that the apoptosis pathway is activated following 6-OHDA-induced ROS generation. For instance, Ramazani *et al*. indicated decreased cell viability, increased ROS, and apoptosis in PC12 cells treated with 6-OHDA (37). Moreover, 6-OHDA caused mitochondrial dysfunctions and enhanced the cleavage of caspase-9 and -3 in the SH-SY5Y cell line (38). Besides, G-CSF could inhibit the activated caspase-3 by 6-OHDA in dopaminergic neurons (39). On the other hand, osthole diminished oxidative stress and inflammatory cytokines in the PD mice model induced by MPTP as well as attenuating LPS-induced microglia cytotoxicity in PC12 and BV-2 cells (32). In this regard, reduced intracellular ROS, caspase-3 activity, and Bax/Bcl2 ratio induced by MPP^+^ through pretreatment with osthole on PC12 cells has been demonstrated (26). In the current study, exposure of cells to 6-OHDA (200 μM) increased intracellular ROS production and elevated caspase-3 cleaved in SH-SY5Y cells. The protective role of osthole (100 μM) against 6-OHDA occurred through attenuating intracellular ROS level and diminished caspase-3 activity, suggesting the anti-oxidant and anti-apoptotic effect of osthole.

Several studies have illustrated that the novel inflammatory signals namely Janus Kinase/Signal Transducers and Activators of Transcription (JAK/STAT), can be activated by LPS, TNF-α, IFN-γ, and IL-6 in the brain (40) and contribute to the pathogenesis of neuroinflammatory diseases (10). For instance, the level of JAK and STAT phosphorylation was rapidly enhanced in BV-2 microglial cells in response to LPS stimulation (41, 42). The α-synuclein accumulation in the brain activated microglial and produced inflammatory cytokines or chemokines through the activation of the JAK/STAT pathway in different models of PD (13). Hence, the administration of JAK1/JAK2 inhibitor, AZD1480, inhibited the activation of JAK, STAT3, and STAT1 in macrophages and microglia cells and suppressed the degeneration of dopaminergic neurons (13). Besides, the neurotoxin MPP^+^ treatment enhanced the STAT1 expression level as well as STAT1 phosphorylation and following apoptosis in cerebellar granule neuron cells (43). Moreover, pyridone 6 as a JAK inhibitor reduced the interferon β neurotoxicity in SH-SY5Y cells through decreased STAT1 and STAT3 phosphorylation as well as apoptotic cell death (44).

Based on the role of the JAK/STAT pathway in brain damage, it is identified that several natural substances such as curcumin and osthole could exhibit protective effects by suppressing JAK/STAT pathways (33, 41, 45). The inflammatory cytokines secretion via LPS-stimulated BV2 cells was decreased because of osthole treatment through Nrf2 and HO1 up-regulation and NFκB signaling pathway inhibition (45). In traumatic brain injury in SH-SY5Y cells, osthole displayed anti-inflammatory and neuroprotective effects due to suppressed apoptosis and NF-κB pathways (46). Osthole also inhibited the proliferation and invasion of gallbladder cancer cells by decreasing the phosphorylation of JAK and STAT3 (33). In addition, 6-OHDA showed a neurotoxicity effect on SH-SY5Y cells by changing the level of STAT3 phosphorylation (47). In this research, results showed the increment of JAK2/STAT3 phosphorylation in SH-SY5Y cells by 6-OHDA treatment, suggesting the role of the JAK2/STAT3 pathway in the PD model. Pretreatment of cells with osthole markedly prevented JAK2 and STAT3 phosphorylation levels in SH-SY5Y cells exposed to 6-OHDA, suggesting osthole has a neuroprotective effect in SH-SY5Y cells by suppressing the JAK2/STAT3 signaling pathway.

The MAPK signaling pathway regulates cell activity including cell viability, apoptosis, as well as inflammation in the CNS (16). ERK and JNK are a part of the MAPKs family that activate through various stimuli like cellular stress and cytokines (14). Previous studies suggested that JNK and ERK play considerable roles in the regulation of cellular processes of PD (9). Transient phosphorylation of ERK participates in enhanced cell survival but sustained ERK activation leads to neuronal and PC12 cell death (48). In this regard, it has been reported that p-ERK1/2 and p-JNK protein levels obviously increased in PD mice (49). A recent study exhibited that oxidative stress could enhance ERK phosphorylation in SH-SY5Y cells (50). Moreover, the MPP^+^ model of PD causes ERK and JNK activation in SH-SY5Y and SH-EP1 cells (51, 52). The neuroblastoma cells’ exposure to MPP^+^ augments α-synuclein, activates ERK, and triggers cell death (53). Besides, α-synuclein provokes inflammation through JNK, ERK, and p38 activation in microglial cells (54). Hadipour *et al*. indicated that 6-OHDA increased ROS production, cell apoptosis, and p-JNK in PC12 cells, which were inverted by pretreatment with betanin (18). Additionally, over-activation of ERK was reported in the striatum of mice in the 6-OHDA-induced PD model (17). 6-OHDA strikingly activated the MAPK pathway through enhancement of JNK, ERK, and p38 phosphorylation in SH-SY5Y cells (38). The ERK phosphorylation and dopaminergic cell death were up-regulated through oxidative stress after rotenone and 6-OHDA treatment, which induced PD progression in experimental animals (55, 56). In line with the studies mentioned above, our results illustrated the enhanced phosphorylation of JNK and ERK in SH-SY5Y cells treated with 6-OHDA. 

A recent study suggested that the endogenous molecules or the plant-derived natural compounds exhibit neuroprotective effects against CNS injury by regulation of the MAPK pathway. In this regard, the beneficial neuroprotective effect of inosine against the rotenone model of PD was shown by ameliorating oxidative stress and pro-inflammatory cytokines along with the inhibition of ERK phosphorylation (56). Another experiment also showed that the primary astrocytes pretreatment by silibinin (a constituent of silymarin) effectively inhibited astroglial activation and decreased ERK and JNK phosphorylation before MPP^+^ treatment in an acute PD model induced by MPTP (57). Furthermore, the administration of C2 ceramide in LPS-stimulated BV2 microglial cells suppressed microglial activation through decreased ROS production and JNK, ERK, and p38 phosphorylation (42). However, different results were reported by Chen *et al*. study. They showed that pretreatment of cells with osthole inhibited the enhancement of p-JNK with no effect on p-p38 but elevated ERK1/2 phosphorylation in neurons that were deficient in oxygen and glucose (27). To date, there is no earlier study, which investigated the osthole effectiveness in PD through the regulation of MAPK activity. For the first time in the current study, pretreatment of cells with osthole could decrease JNK and ERK phosphorylation in SH-SY5Y cells treated with 6-OHDA.

## Conclusion

In summary, osthole could be able to diminish 6-OHDA cytotoxicity in SH-SY5Y cells, in part, to be mediated through the inhibition of ROS production, decreased apoptosis, and reduction of JAK/STAT and MAPK activity, which are important mechanisms in 6-OHDA neurotoxicity (Figure 6).

## Authors’ Contributions

S B contributed to data analysis, writing, and reviewing. PH performed data handling, data analysis, and data presentation. G K designed the experiments, supervised, validated the study, and reviewed. Z TN analyzed the data, and validated the study. SM designed experiments, provided study materials and equipment, supervised, validated the study, and reviewed. 

## Funding Informwtion

This paper was financially supported by the Vice Chancellor of Research, Mashhad University of Medical Sciences, Mashhad, Iran (Grant Number: 970912).

## Conflicts of Interest

The authors declare no conflicts of interest.
